# Association of MCP-4, NRTN, and PD-L1 with the risk of hepatic fibrosis: A Mendelian randomization study

**DOI:** 10.1097/MD.0000000000039655

**Published:** 2024-09-06

**Authors:** Liqun Li, Jing Yan, Qian Liu, Laian Ge, Yifeng Pan, Bingjie Han, Chunmei Wang, Xiaomei Tang, Lijian Liu, Sheng Xie

**Affiliations:** a First Affiliated Hospital of Guangxi University of Chinese Medicine, Nanning City, China; b Graduate School of Guangxi University of Chinese Medicine, Nanning City, China; c Affiliated Hospital of Jiangxi University of Chinese Medicine, Nanchang City, China; d Eighth Clinical Medical College of Guangzhou University of Chinese Medicine, Foshan City, China.

**Keywords:** bidirectional, blood metabolites, circulating inflammatory cytokines, hepatic fibrosis, Mendelian randomization

## Abstract

Previous studies have confirmed the affiliation between specific inflammatory cytokines and Hepatic fibrosis (HF); however, contradictions remain in the causality. The study implemented a bidirectional two-sample Mendelian randomization (MR) analysis with published statistics derived from Genome-wide Association Studies (GWAS) to investigate casualties between inflammatory cytokines and HF. Additionally, MR analysis was also introduced to consider if 1400 blood metabolites act as the key mediators in this process. Single nucleotide polymorphisms (SNPs) with strong correlations to inflammatory factors were selected for multiple MR analyses in this study. The inverse variance weighted method (IVW) was chosen as the principal analysis, and the others as the supportive. Besides, sensitivity tests were involved to identify potential heterogeneity and pleiotropic level. IVW methods revealed that a relatively high level of prediction-based monocyte chemoattractant protein-4 (MCP-4) (95% CI: 1.014–3.336, *P* = .045), along with neurturin (NRTN) (95% CI: 1.204–4.004, *P* = .010), may increase the risk of HF; while programmed cell death 1 ligand 1 (PD-L1) (95% CI: 0.223–0.928, *P* = .030), showed a protective effect on HF. No significant statistical differences were detected on any other inflammatory cytokines, nor did the impact of HF genetic predisposition on the 91 circulating inflammatory cytokines-related characteristics.

## 1. Introduction

As one of the world’s primary causes of disease, it has been predicted that the global burden of HF may witness a further increase in the coming years.^[1]^ HF is regarded as hepatic histopathological changes caused by the excessive accumulation of extracellular matrix, which results from cell damage induced by factors such as excessive drinking, viral hepatitis, lipid accumulation, thereby leading to fibrous scarring on the liver.^[2]^ The prognosis of HF is generally unfavorable due to its insidious early onset and long process.^[3]^ Without timely and effective intervention, irreversible hepatic cirrhosis (HC) and even hepatic cancer (HCC) may develop.^[4]^ According to epidemiological investigations, the morbidity of HF in the population progression stage reaches 2.85% in China;^[5]^ as the terminal stage of progressive HF, HC has an impact on 1% to 2% of the global population, causing over 10,000 deaths per year.^[6,7]^ A survey showed that anti-inflammatory diet treatment plans may effectively hinder the progression from chronic hepatic disease to end-stage HF in American adults.^[8]^ Since the pathogenesis of HF remains unclear at present, it is obstructive to implement comprehensive suppression of the fibrosing progress, leading to clinical unsatisfactory. The key to treating HF is to heal the fibrosis of liver tissue, which may indicate the importance of anti-inflammatory, liver-protective medications.^[9,10]^ However, due to the current absence of chemical or biological agents specifically targeting HF, with proven and acceptive therapeutic efficacy, the risks of HC and HCC are, to some extent, increased.^[11]^ Therefore, there is an urgent need to investigate the pathogenesis of HF to seek improved strategies for the prevention and treatment, thereby minimizing the possible damage.

Although the exact mechanism of HF remains elusive currently, studies have demonstrated the complex interactions between HF and inflammatory cytokines. The relationship between HF and certain inflammatory factors remains highly controversial, for example, some researchers believe that elevated expression of programmed cell death 1 ligand 1 (PD-L1) gene may help relieve HF;^[12]^ while other studies showed that PD-L1 can increase the risk of HF.^[13]^ In addition, HF is directly related to the expression of C-C motif chemokine 4 (CCL4), Interleukin-8 (IL-8) and other genes.^[14]^ At the same time, the pathogenesis of HF produces a lot of inflammatory mediators, such as PD-L1, CCL4, CCL2, IL-8, etc.^[15]^ However, the argument about whether inflammatory factors are the cause of HF or a consequence of its progression remains controversial. Although observative research have attempted to elucidate the casualties between inflammatory cytokines and HF, unexpected confounding factors or reverse causalities may lead to bias, resulting in blurred relationships. Therefore, it is necessary to clarify the causal connection between inflammatory cytokines and HF based on a reliable method.

Utilizing genetic variations as instrumental variables (IVs), Mendelian randomization (MR) evaluates the causal association between exposure factors and disease outcomes.^[16]^ It can not only minimize the disturbance from confounding factors in traditional observational studies but also avoid possible reverse causalities.^[17]^ Although widely applied to explore disease onset risks, MR has not yet been implemented in investigations for the casualties between inflammatory cytokines and HF, as well as the mediating role of metabolites. Therefore, in this study, genetic instruments were utilized as alternatives to circling inflammatory cytokines to evaluate their causal effect on the development of HF, and to determine whether 1400 metabolites mediate the whole process. More genetic-biological understanding may be provided by elucidating these casualties, as well as paving a new effective way for the intervention of HF.

## 2. Materials and methods

### 2.1. Research design

**Figure 1.**
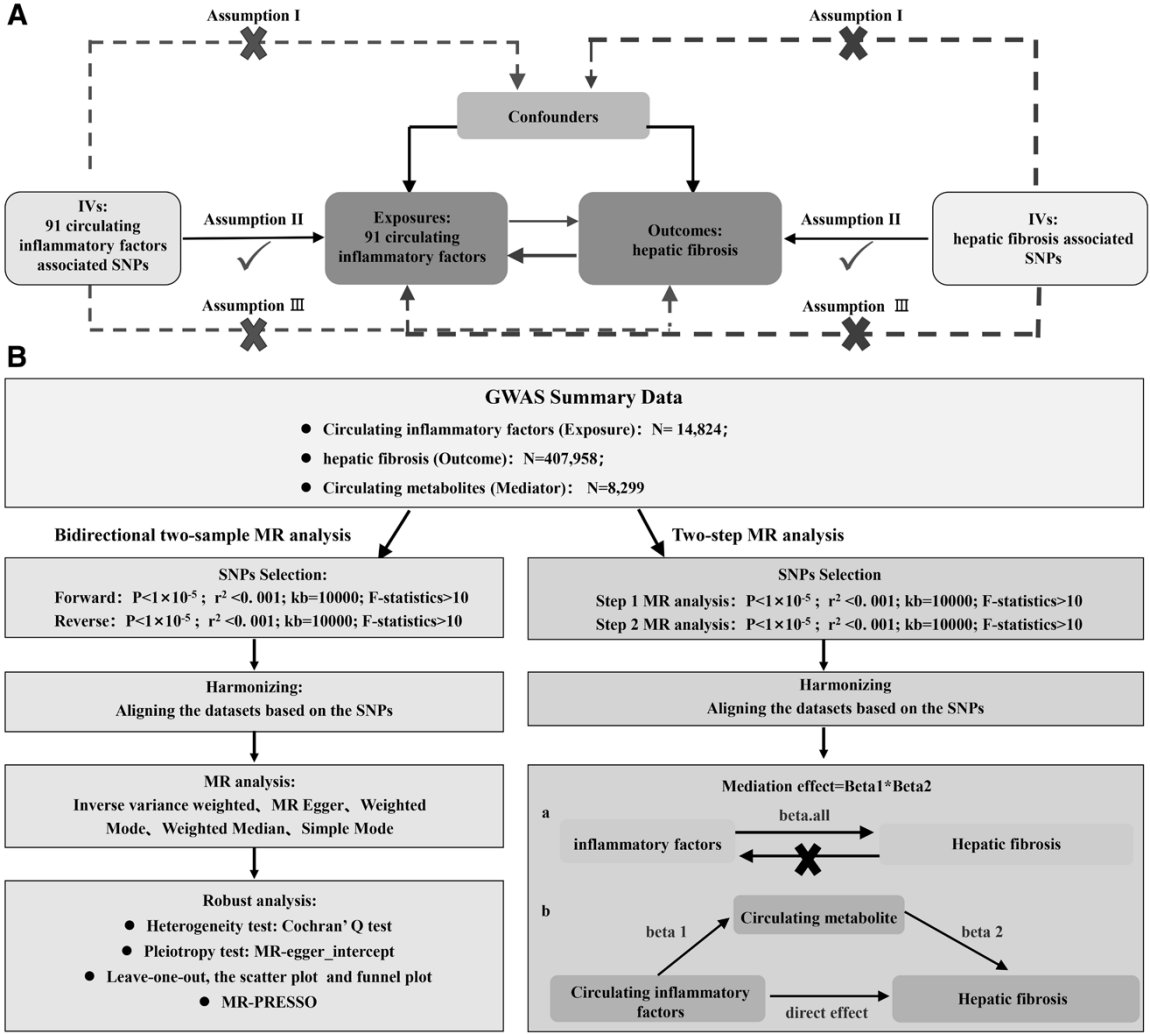
Assumptions and study design of the bidirectional MR study of 91 circulating inflammatory factors in association with HF. IVs = instrumental variables, SNPs = single nucleotide polymorphisms.

The design of MR is demonstrated in Figure 1. Bidirectional MR relies on three main assumptions. Genetic variations are not associated with confounding factors; genetic variations selected as IVs correlate strongly with exposure; and generic variations solely influence outcome via exposure.^[18]^ This study exploited summary data from known genome-wide association studies (GWAS) of 91 inflammatory factors and HF. Firstly, casualties between inflammatory factors and HF were inferred according to the selection of genetic variations. Subsequently HF-related genetic variations were then selected to deduce casualties between HF and the 91 circulating inflammatory cytokines. Since this study is based on summarized genetic data from the GWAS database, no extra ethical approval is required. The study was designed and drafted according to the principles of the MR report, *Strengthening the Reporting of Observational Studies in Epidemiology–Mendelian randomization* (STROBE-MR).^[19]^

### 2.2. Data source

The GWAS summary data of HF came from FinnGen (https://www.finngen.fi/en).^[20]^ As one of the most sizable genome research databases at present, FinnGen aims to improve human health via genome research and eventually achieve new therapeutic targets and diagnosing approaches for multiple diseases. The HF-GWAS data, identified by the ID “finngen_R10_FIBROLIV.gz,” encompasses 407,958 participants, including 157 HF cases and 407,801 controls (Table S1, Supplemental Digital Content, http://links.lww.com/MD/N541).

Data of inflammatory factors were originate from the GWAS database, including 14,824 European participants. The integrated GWAS data of this research is available in the BI-GWAS catalog (Table S2, Supplemental Digital Content, http://links.lww.com/MD/N541). Related details can be found in the original publication.^[21]^

The data concerning 1400 blood metabolites came from a GWAS cohort with 8299 individuals,^[22]^ and the complete dataset can be downloaded from the NHGRI-EBI GWAS Catalog (https://www.ebi.ac.uk/gwas/) (Table S3, Supplemental Digital Content, http://links.lww.com/MD/N541). Two-sample MR analysis was then applied to the obtained GWAS data to infer the causal association between HF and circulating inflammatory cytokines. Since the participants involved in our study are all from the European, racial bias was avoided to some extent.

### 2.3. Selection of instrumental variations

A serious of methods were performed to filter out effective SNPs that conform to the three core MR assumptions. First, we settled the genome-wide significance threshold as *P* < 1e-05 of circulating inflammatory cytokines, HF and metabolites to identify strongly related SNPs. Second, to ensure the independence of these SNPs and eliminate bias caused by linkage disequilibrium (LD), the parameter indicated the exclusion of LD was set as clump kb = 10,000 and clump *r*^2^ = 0.001. Finally, F-statistics (F > 10) was introduced to evaluate the strength of SNPs: F = *R*^2^ × (n − 2)/(1 – *R*^2^). *R*^2^ represents the proportion of trait variance illustrated via SNPs, and N indicates the size of the sample with the specific characteristic.^[23]^ Similarly, the formula *R* = 2 × EAF × (1 − EAF) × β was introduced to determine the *R* value, meanwhile reducing bias caused by weak instrumental variations. Characteristics of SNPs involved in this study were listed in detail in the Tables S1–3, Supplemental Digital Content, http://links.lww.com/MD/N541.

### 2.4. Mendelian randomization analysis

With the obtained related GWAS data, MR analysis was applied to study the casualties between inflammatory factors and HF. The inverse variance weighting (IVW) analysis served as the major method for predicting MR value. As a robust Mendelian randomization analysis, IVW can provide concise estimates.^[24]^ Besides, secondary analyses were also implemented via MR-Egger, Weighted Mode, Weighted Median, and Simple Mode methods.^[25]^ MR-Egger intercept test was utilized to identify and calibrate pleiotropy.^[26]^ Cochran’s Q analysis was introduced to assess whether heterogeneity exists between SNPs selected by every circulating inflammatory cytokine, if so, the IVW random-effect model would be utilized as the principal analytical approach.^[27]^ Mendelian randomization pleiotropy residual sum and outlier (MR-PRESSO) were applied to identify possible outliers, which would be excluded before initiating a new analysis to examine and correct for horizontal pleiotropy.^[26]^ Stabilities of outcomes and outliers produced above were tested via Leave-One-Out (LOO) sensitivity analysis, Scatter Plot, and Funnel Plot. All the analyses were performed with Ieugwasr, gwasglue, gwasvcf, ieugwasr, MRInstrument, and software package in R (Ver 4.3.3). The threshold of statistical significance was *P* < .05.

### 2.5. Mediation analysis

Mediation analysis was further demonstrated through two-step MR analysis to assess the involvement of metabolites in the casualties between inflammatory cytokines and HF, which can be diversified into indirect (mediated) effects and direct (non-mediated) effects.^[28]^ The first step is to perform MR on inflammatory cytokines and metabolites (beta 1); the second is to perform MR on metabolites and HF (beta 2); then perform MR on inflammatory cytokines and HF (beta all); finally, determine the mediation effect. The screening condition was set at *P* < .05 in the analysis to ensure the adequacy of metabolites that passed the screening.

## 3. Results

### 3.1. The role of circling inflammatory cytokines in HF progress

**Figure 2.**
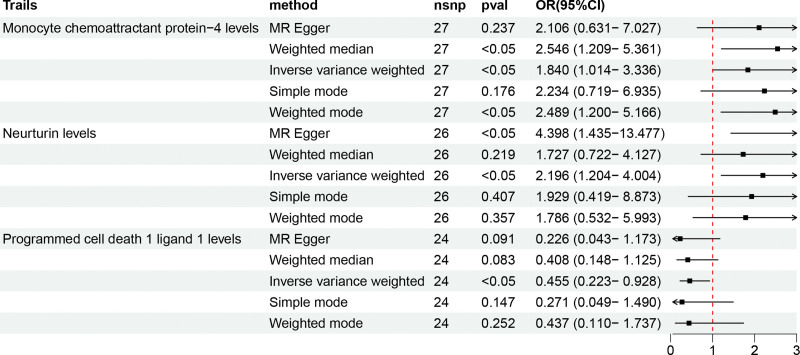
Forest plots of the causal association between MCP-4, NRTN, and PD-L1 and HF in the result in the forward MR analysis. MCP-4 = monocyte chemoattractant protein-4, NRTN = neurturin, nsnp = the number of single nucleotide polymorphism, PD-L1 = programmed cell death 1 ligand 1.

**Figure 3.**
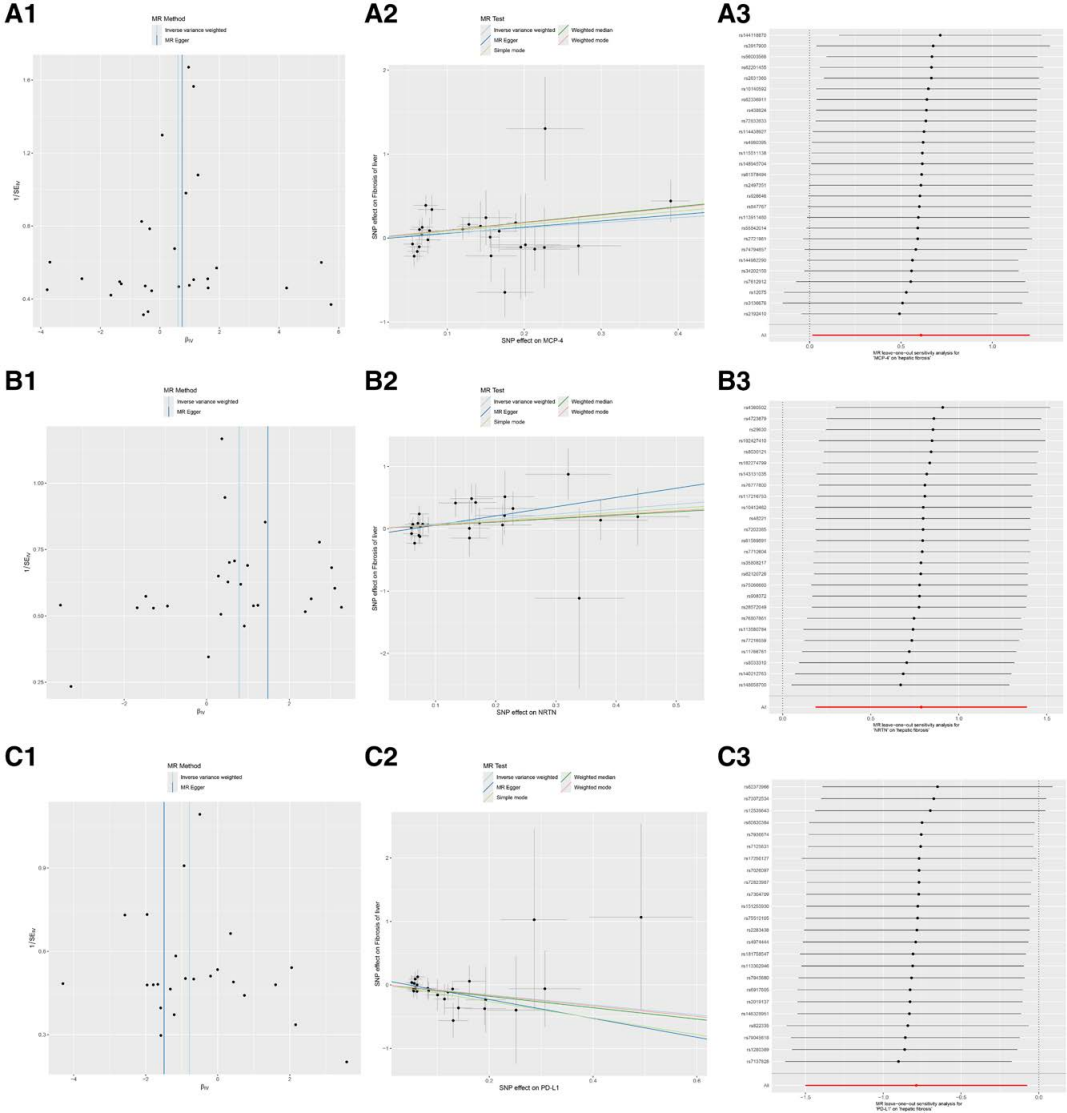
Scatter plot, funnel plot, and LOO analysis of the causal association between MCP-4, NRTN, and PD-L1 and HF. (A1), (A2), (A3) Scatter plot, funnel plot, and LOO analysis of the causal association between MCP-4 and HF. (B1), (B2), (B3) Scatter plot, funnel plot, and LOO analysis of the causal association between NRTN and HF. (C1), (C2), (C3) Scatter plot, funnel plot, and LOO analysis of the causal association between PD-L1 and HF. MCP-4 = monocyte chemoattractant protein-4, NRTN = neurturin, PD-L1 = programmed cell death 1 ligand 1, SNP = single nucleotide polymorphism.

After screening with the methods above, all the F-statistics of selected SNPs are within the range of 19.510-3549.334 (Table S4, Supplemental Digital Content, http://links.lww.com/MD/N541), which indicates the robustness of SNPs involved in this study. Then the SNPs were utilized for conducting a causal analysis between inflammatory cytokines and HF. The Table S5-A, Supplemental Digital Content, http://links.lww.com/MD/N541 showed MR outcomes with different methods. Specifically, primary results of IVW revealed that two inflammatory cytokines were associated positively with HF in a causal manner, including monocyte chemoattractant protein-4 (MCP-4) (OR = 1.840, 95% CI: 1.014–3.336), *P* = .045) and neurturin (NRTN) (OR = 2.196, 95% CI: 1.204–4.004, *P* = .010); while the causal relationship between Programmed cell death 1 ligand 1 (PD-L1) (OR = 0.455, 95% CI: 0.223–0.928, *P* = .030) and HF was found to be negative (Fig. 2). Cochran’s Q test found heterogeneity in the outcomes of none of the three inflammatory cytokines, and the MR-Egger intercept test did not show evidence of pleiotropy either. Besides, MR-PRESSO demonstrated that there was no horizontal pleiotropy presented in this MR analysis (Table S5-B, Supplemental Digital Content, http://links.lww.com/MD/N541). Moreover, the scatter plot and funnel plot both conformed to the characteristics of the positive outcomes provided by the MR analysis, and the LOO analysis proved the robustness of the outcome (Fig. 3).

MR-Egger analysis demonstrated the relationships between C-X-C motif chemokine 9, interleukin-5, leukemia inhibitory factor receptor, and neurotrophin-3, with the onset of HF; Weighed median test found the association between Protein S100-A12 and HF risk, but IVW did not show evidence of the possible relationship between this five circulating inflammatory cytokines and HF (Supplementary Table S5-A, http://links.lww.com/MD/N541). Therefore, mainly based on IVW analysis, this study believed that apart from MCP-4, NRTN, and PD-L1, the other 88 inflammatory cytokines showed no correlations with HF risks.

### 3.2. Effect of HF on inflammatory cytokines

As demonstrated in Table S6, Supplemental Digital Content, http://links.lww.com/MD/N541, the F-statistics in the reverse MR analysis ranged from 20.635 to 20.821, which indicated the robustness of all the selected instrumental SNPs. The outcomes of MR analysis showed that the genetic predisposition of HF has no significant impact on the relevant characteristics of 91 circulating inflammatory cytokines (Table S7-A, Supplemental Digital Content, http://links.lww.com/MD/N541). Meanwhile, the MR-Egger intercept test detected no pleiotropy, which further proved the reliability of the research outcomes (Table S7-B, Supplemental Digital Content, http://links.lww.com/MD/N541).

### 3.3. Effect of inflammatory cytokines on metabolites

All the selected metabolites-related SNPs were robust instruments with F-statistics ranging between 19.503 and 2297.785 (Table S3, Supplemental Digital Content, http://links.lww.com/MD/N541). IVW was applied to determine the causal impacts of the two inflammatory cytokines on the five metabolites (Table S8-A, Supplemental Digital Content, http://links.lww.com/MD/N541).

As shown in Table 1, NRTN was associated positively with the level of laurate (12:0) (OR = 1.110, 95% CI: 1.007–1.223, *P* = .035); while negatively with 1-linoleoyl-GPE (18:2) (OR = 0.881, 95% CI: 0.799–0.971, *P* = .011), adenosine 5’-diphosphate (ADP) to tyrosine ratio (OR = 0.821, 95% CI: 0.721–0.935, *P* = .003), and hypotaurine to cysteine ratio (OR = 0.902, 95% CI: 0.815–0.999, *P* = .047). Besides, PD-L1 was also found positively correlated with the level of 1-palmitoyl-2-linoleoyl-GPI (16:0/18:2) (OR = 1.110, 95% CI: 1.015–1.213, *P* = .022). Cochran’s Q test found no significant heterogeneity in the outcomes above, and the MR-Egger intercept test and MR-PRESSO test demonstrated no evidence of pleiotropy either (Table S8-B, Supplemental Digital Content, http://links.lww.com/MD/N541).

**Table 1:** MR analysis results for causal effects of inflammatory factor levels on mediators circulating metabolites (*P *< .05).

Inflammatory factor levels	Circulating metabolites levels	MR results			
		Beta	OR	95% CI	*P* value
NRTN	1-linoleoyl-GPE (18:2) levels	−0.127	0.881	0.799–0.971	.011
	Laurate (12:0) levels	0.104	1.110	1.007–1.223	.035
	Adenosine 5’-diphosphate (ADP) to tyrosine ratio	−0.197	0.821	0.721–0.935	.003
	Hypotaurine to cysteine ratio	−0.103	0.902	0.815–0.999	.047
PD-L1	1-palmitoyl-2-linoleoyl-GPI (16:0/18:2) levels	0.104	1.110	1.015–1.213	.022

CI = confidence interval, NRTN = neurturin, OR = odds ratio, PD-L1 = programmed cell death 1 ligand 1.

### 3.4. Effect of metabolites on HF

46 metabolites were detected as significantly related to HF in the causal analysis between 1400 metabolites and HF afterward (Table S9-A, Supplemental Digital Content, http://links.lww.com/MD/N541). As shown in Table 2, among the 46 robust casualties, 25 metabolites showed a positive correlation with HF, including 1-linoleoyl-GPE (18:2) (OR = 1.789, 95% CI: 1.190–2.691, *P* = .005), 1-palmitoyl-2-linoleoyl-GPI (16:0/18:2) (OR = 2.093, 95% CI: 1.219–3.594, *P* = .007), and adenosine 5’-diphosphate (ADP) to tyrosine ratio (OR = 1.484, 95% CI: 1.002–2.197, *P* = .049); while 21 metabolites exhibited a negative association with HF, such Hypotaurine to cysteine ratio (OR = 0.508, 95% CI: 0.274–0.942, *P* = .031). Cochran’s Q test found no evidence of significant heterogeneity, and the absence of statistical significance in the MR-Egger intercept test and MR-PRESSO test both indicated that there was no pleiotropy in the outcomes (Table S9-B, Supplemental Digital Content, http://links.lww.com/MD/N541).

**Table 2:** Detailed results of MR analysis for causal effects of circulating metabolites levels on HF (*P <* .05).

Circulating metabolites levels	nsnp	MR results				
		Beta	SE	OR	95% CI	*P* value
2-hydroxyoctanoate levels	22	−0.477	0.214	0.621	0.408–0.944	.026
Tauro-beta-muricholate levels	26	−0.342	0.152	0.711	0.528–0.957	.024
Stearidonate (18:4n3) levels	26	−0.790	0.317	0.454	0.244–0.844	.013
Gamma-glutamylmethionine levels	25	−0.681	0.298	0.506	0.282–0.907	.022
3-(3-amino-3-carboxypropyl)uridine levels	17	−0.757	0.375	0.469	0.225–0.979	.044
2-hydroxy-3-methylvalerate levels	30	−0.556	0.274	0.573	0.335–0.981	.042
**1-linoleoyl-GPE (18:2) levels**	**33**	**0.582**	**0.208**	**1.789**	**1.190–2.691**	**.005**
1-palmitoyl-2-linoleoyl-GPE (16:0/18:2) levels	25	0.403	0.163	1.496	1.086–2.060	.014
2-hydroxyglutarate levels	28	−0.572	0.273	0.565	0.331–0.964	.036
S-methylcysteine sulfoxide levels	21	0.890	0.397	2.434	1.118–5.299	.025
N-acetylcarnosine levels	28	−0.381	0.187	0.683	0.473–0.986	.042
1-palmitoyl-2-palmitoleoyl-gpc (16:0/16:1) levels	23	0.775	0.302	2.170	1.202–3.918	.010
1-palmitoyl-2-docosahexaenoyl-gpc (16:0/22:6) levels	23	−0.773	0.277	0.461	0.268–0.794	.005
1-stearoyl-2-linoleoyl-GPI (18:0/18:2) levels	28	0.524	0.239	1.689	1.058–2.696	.028
1-(1-enyl-palmitoyl)-2-linoleoyl-GPE (*P*-16:0/18:2) levels	22	0.681	0.292	1.976	1.114–3.505	.020
1-oleoyl-2-linoleoyl-GPE (18:1/18:2) levels	33	0.591	0.177	1.807	1.277–2.556	.001
N-stearoyl-sphingadienine (d18:2/18:0) levels	21	−0.419	0.205	0.658	0.440–0.983	.041
2-furoylcarnitine levels	22	0.981	0.277	2.666	1.548–4.590	.000
Carotene diol (3) levels	27	0.702	0.248	2.018	1.241–3.280	.005
Pentose acid levels	22	0.695	0.349	2.004	1.012–3.968	.046
Methyl indole-3-acetate levels	22	−0.674	0.288	0.509	0.289–0.897	.019
**1-palmitoyl-2-linoleoyl-GPI (16:0/18:2) levels**	**24**	**0.739**	**0.276**	**2.093**	**1.219–3.594**	**.007**
N-formylmethionine levels	21	−0.765	0.336	0.465	0.241–0.900	.023
2-hydroxyhippurate (salicylurate) levels	26	0.688	0.321	1.989	1.060–3.731	.032
Cystine levels	18	−0.821	0.408	0.440	0.198–0.979	.044
X-11632 levels	25	−0.604	0.296	0.547	0.306–0.976	.041
X-12544 levels	23	0.828	0.282	2.289	1.317–3.978	.003
X-17010 levels	19	−0.707	0.343	0.493	0.252–0.966	.039
X-24344 levels	16	0.792	0.287	2.208	1.258–3.875	.006
X-24337 levels	28	0.491	0.236	1.634	1.028–2.597	.038
X-25957 levels	20	0.815	0.367	2.260	1.100–4.643	.026
X-25420 levels	15	−1.052	0.282	0.349	0.201–0.606	.000
1-palmitoyl-2-arachidonoyl-gpc (16:0/20:4n6) levels	31	−0.326	0.158	0.722	0.529–0.984	.039
X-19141 levels	25	0.493	0.236	1.637	1.030–2.600	.037
**Adenosine 5’-diphosphate (ADP) to tyrosine ratio**	**22**	**0.395**	**0.200**	**1.484**	**1.002–2.197**	**.049**
Cortisone to cortisol ratio	20	−0.689	0.345	0.502	0.255–0.988	.046
Methionine to methionine sulfoxide ratio	27	0.628	0.303	1.873	1.034–3.391	.038
Oleoyl-linoleoyl-glycerol (18:1 to 18:2) [2] to linoleoyl-arachidonoyl-glycerol (18:2 to 20:4) [2] ratio	28	0.413	0.181	1.511	1.061–2.153	.022
Adenosine 5’-monophosphate (AMP) to citrate ratio	23	−0.658	0.328	0.518	0.272–0.985	.045
**Hypotaurine to cysteine ratio**	**34**	**−0.678**	**0.315**	**0.508**	**0.274–0.942**	**.031**
Retinol (Vitamin A) to linoleoyl-arachidonoyl-glycerol (18:2 to 20:4) [1] ratio	24	0.573	0.235	1.774	1.118–2.813	.015
Phosphate to EDTA ratio	20	0.860	0.384	2.363	1.112–5.019	.025
Cytidine to N-acetylneuraminate ratio	28	0.687	0.289	1.988	1.129–3.502	.017
Phosphoethanolamine to choline ratio	22	0.762	0.364	2.143	1.049–4.376	.036
Caffeine to linoleate (18:2n6) ratio	14	−0.740	0.334	0.477	0.248–0.919	.027
Salicylate to taurocholate ratio	23	0.961	0.349	2.615	1.320–5.178	.006

CI = confidence interval, nsnp = the number of single nucleotide polymorphism, OR = odds ratio, SE = standard error.

1-linoleoyl-GPE (18:2) levels, 1-palmitoyl-2-linoleoyl-GPI (16:0/18:2) levels, and adenosine 5′-diphosphate (ADP) to tyrosine ratio, hypotaurine to cysteine ratio had *P*-values <0.05, indicating that these 4 circulating metabolites are closely related to the development of hepatic fibrosis.

### 3.5. Mediation effect

In the mediation analysis of 1400 blood metabolites, three metabolites were identified as possible mediators of the increasing inflammatory cytokine NRTN and HF risk. 1 − linoleoyl − GPE (18:2) demonstrated a mediation effect of -0.0737 (95% CI: −0.15, 0.00287); Adenosine 5’-diphosphate (ADP) to tyrosine ratio showed a mediation effect of −0.0777 (95% CI: −0.17, 0.0151); Hypotaurine to cysteine ratio exhibited a mediation effect of 0.0699 (95% CI: −0.024, 0.164). Besides, metabolites 1-palmitoyl-2-linoleoyl-GPI (16:0/18:2), showing a mediation effect of 0.0768 (95% CI: −0.00982, 0.163), might also mediate the growing of cytokine PD-L1 level and provide protection against HF. Even the metabolites above showed effects relating to changes in NRTN and PD-L1, according to *P* value (*P* > .05), however, none of them were considered statistically significant (Table S10, Supplemental Digital Content, http://links.lww.com/MD/N541).

### 3.6. Sensitivity analysis

Multiple sensitivity analyses were applied to evaluate whether the outcomes were heterogeneous. The results of these analyses were demonstrated in Table S5-B, Supplemental Digital Content, http://links.lww.com/MD/N541, S7-B, http://links.lww.com/MD/N541, S8-B, http://links.lww.com/MD/N541, and S9-B, http://links.lww.com/MD/N541. Although significant heterogeneity was detected in some of the results, after calibration with random effect models, no substantial changes were found in IVW outcomes. Besides, the MR-Egger intercept test was applied to examine potential pleiotropy and the MR-PRESSO global test was utilized to solve possible heterogeneity, whose outcomes showed no evidence of pleiotropy in the positive results. The consistency of sensitivity analysis enhanced the robustness of the outcomes of this study.

## 4. Discussion

Utilizing the largest scale of GWAS data for MR analysis so far, this research evaluated the causalities between 91 inflammatory cytokines and HF risks and analyzed whether the 1400 blood metabolites served as mediators in these correlations. The results of the study demonstrated that the levels of MCP-4 and NRTN were associated positively with HF risk, while that of PD-L1 level displayed an opposite relationship. Besides, the genetic disposition of HF showed no evidence of causality with other 88 inflammatory cytokines. Meanwhile, the outcomes of the mediation analysis demonstrated that 1 − linoleoyl − GPE (18:2), Adenosine 5’−diphosphate (ADP) to tyrosine ratio and Hypotaurine to cysteine ratio may show a potential mediating effect on the increase in HF risk induced by elevated NRTN levels; while 1-palmitoyl-2-linoleoyl-GPI (16:0/18:2) may mediate the increase in PD-L1 levels leading to a decrease in HF risk. Noticeably, the robustness of these outcomes was supported by sensitivity analysis. To conclude, the findings of this research provided valuable insights into the role of inflammatory factors and their metabolites in HF prediction & treatments.

Although the pathogenesis of HF remains unclear so far, it has been proven that inflammatory factors play a crucial role in adjusting the process. The pathological generation of certain inflammatory cytokines may trigger an abnormal immune response in individuals with HF, thereby inducing or even aggravating the inflammatory cascade. Studies have confirmed that HF is the outcome of the mediation of inflammatory cytokines.^[29]^ MCP-4, alias CCL13, is a significant chemokine that mediates inflammation. MCP-4 mainly induces the production of proinflammatory factors and the expression of cell adhesion molecules in endothelial and epithelial cells.^[30]^ Besides, MCP-4 may trigger immunomodulatory responses by adjusting the epithelial and endothelial cell functions.^[31]^ In humans, MCP-4 plays a key role in various chronic inflammatory responses by modulating immune cell infiltration.^[32]^ These findings indirectly support the results of this study that prediction-based, higher MCP-4 levels may increase the HF risk. This association may be attributed to MCP-4 inducing the generation of proinflammatory cytokines in endothelial and epithelial cells, triggering immunomodulatory responses, thereby accelerating the production of profibrotic factors and mobilization of immune cells to the site of damage, ultimately involving in the process of inflammatory-damage induced hepatic fibrosis.^[33]^

MR analysis outcomes showed that prediction-based NRTN level was relevant to an increased risk of HF. Both sensitivity analysis and pleiotropy analysis indicated the robustness and non-reversal of causality. As an important member of the glial-derived neurotrophic factor (GDNF) family, NRTN participates in the modulation of the body’s immune and inflammatory responses.^[34]^ The expression level of GDNF witnessed an increase along with HF progression.^[35]^ NRTN can participate in the development of liver diseases via the PI3K/AKT/mTOR pathway.^[36]^ Experiments showed that exosomes originated from human adipose-derived mesenchymal stem cells (MSCs) can inhibit the PI3K/AKT/mTOR pathway to reduce liver inflammation, promote choline metabolism, and ultimately improve liver fibrosis.^[37]^ The evidence above provided a possible explanation for the outcomes of the MR analysis in this research, which is that NRTN may trigger MSCs inflammatory response and choline metabolism abnormality via PI3K/AKT/mTOR pathway, thereby increasing HF risks.

Previous research found that PD-L1 may increase HF risks.^[13]^ However, this study demonstrated controversial conclusions. While the mentioned research utilized mouse stem cells (AML-12) as objectives, this research mainly involved genetic variations of the European population group as IVs. Genes of mice were considered highly similar to those of humans; however, they are not able to serve as a full simulation, which may be the major cause of the outcome conflicts. By performing a MR analysis, this study minimized the impact of confounding variables and reverse causality, thereby strengthening the robustness of results. PD-L1 is known to be a crucial signal for regulating immune responses. It can modulate the progression of HF by reprogramming the functions of hepatic cells, dendritic cells (DCs), and T cells.^[38]^ The expression of PD-L1 is widely observed in various hematopoietic cells, including T cells, DCs, epithelial cells, endothelial cells, fibroblasts, etc., playing a crucial part of HF pathogenesis.^[39,40]^ Studies found that aiming at the PI3K/AKT/FoxO1 signaling pathway can inhibit the maturation of dendritic cells, thereby hindering the activation of CD8 + T cells and HSCs, and alleviating hepatic damage and HF inflammation.^[12]^ The research evidence provided possible explanations for MR outcomes demonstrated in this study, which is that PD-L1 may suppress the activation of CD8 + T cells and HSCs through the PI3K/AKT/FoxO1 pathway, consequently reducing HF risks.

Our MR results proved that MCP-4 and NRTN are potential latent risks of HF. PD-L1 served as a potential protective factor of HF and this association was regarded as irreversible. However, there is a lack of studies revealing the direct correlations between these inflammatory cytokines and HF risks by far. This underscores the need for further excavation of this subject matter to demonstrate a comprehensive understanding of the specific role that the mentioned circulating inflammatory cytokines played in HF process, thereby bolstering the foundation for potential therapeutic strategies.

Previous studies highlighted the critical roles of IL18, CXCL10, IL7, CXCL9, and CCL2 in the process of hepatic fibrosis and related inflammatory defects;^[41]^ inflammatory cytokines such as beta-nerve growth factor, Caspase 8 may hinder the process of hepatic fibrosis by enhancing hepatic cell apoptosis.^[42,43]^ Other studies found that plasma levels of CCL20, CCL4, and IL10RB may be important factors that mediate the progression from nonalcoholic fatty liver disease to HF.^[44,45]^ Clinical evidence indicated the significance of IL-6, TNF-α, MIP-1β in determining the clinical stage of HF.^[46]^ Moreover, the escalating levels of plasma IL-6 have been identified as a key indicator of HF progression. However, the MR results demonstrated in this study, forward or reverse, neither indicated any substantial causalities between these circulating inflammatory cytokines and HF risks. This conflict may result from confounding factors including population groups, geographical locations, climatic conditions, age, and gender. Since the potential biofunction of these inflammatory cytokines and the current bottleneck in treating HF, further investigations were necessary to fully study the exact causalities between these inflammatory cytokines and HF risks.

Mediation analysis involved in this study demonstrated that although 1 − linoleoyl − GPE (18:2), Adenosine 5’-diphosphate (ADP) to tyrosine ratio, Hypotaurine to cysteine ratio were considered correlated to an increase in NRTN levels, and 1-palmitoyl-2-linoleoyl-GPI (16:0/18:2) was related to increasing PD-L1 level, but the evidence for the mediating role of these 4 metabolites in the potential pathway linking NRTN, PD-L1, and HF risks was deemed insufficient. This may be due to an inadequate sample size, unobvious mediating effects, or other confounding factors that were not yet taken into consideration. Although the current analysis is still far from establishing significance for these metabolites as mediators, however, possibilities still exist as they play certain roles in the biological process between NRTN, PD-L1, and HF risks. Further studies may continue the investigation of the potential mechanisms of these metabolites and possible utilizations of them as targets for HF prevention or treatments.

This study enjoys several strengths, mainly the MR design and mediation analysis. Utilizing GWAS summary data on the largest scale so far, this research employed MR analysis to evaluate the causalities between 91 inflammatory factors and HF risks, as well as the role of 1400 metabolites as mediators in this process. Compared to observational researches, MR analysis is less subject to unknown confounding factors and reverse causalities. Randomized controlled trials (RCT), often applied for determining causalities between exposure and outcomes, are both timely and economically costive, which is a practical challenge that should be considered carefully. MR analysis may serve as an effective alternative to RCTs, for it can minimize confounding factors and reverse causalities by applying IVs related to exposures and outcomes to evaluate causalities.^[47]^ Besides, rigorous outlier evaluation and wide sensitivity analysis were applied to enhance the robustness of the study outcomes, thereby improving their reliability. Based on the findings of this study, further studies could be conducted to investigate the intervention of these positive circulating inflammatory cytokines in HF, aiming to precisely prevent and treat HF, further enhancing the clinical efficacy of the disease and improving prognosis.

However, limitations also remain in this research. First, since the data involved all came from the European population group, obstructions may occur when attempting to popularize the outcomes of this research to other population groups such as Americans or Asians. Second, due to the utilization of GWAS summary data, it was less likely to provide stratified analysis according to other parameters including age, gender, geography, and climate. Third, although pleiotropy analysis and MR-PRESSO method were employed to minimize possible confounding factors, complete elimination was not feasible Lastly, potential downstream mechanisms of circulating inflammatory cytokines and HF pathogenesis were not yet involved in the study.

## 5. Conclusion

With the application of MR analysis, the study supplies convincing evidence for the casualties between inflammatory cytokines and HF. We confirmed that MCP-4 and NRTN may increase the risk of HF while PD-L1 may have the opposite effect. Besides, some blood metabolites were detected correlated to the increase of NRTN and PD-L1, however, the current evidence is insufficient to prove their potential role as mediators of the pathway between NRTN, PD-L1 and HF risk. The findings of this research help deepen the comprehension of HF pathogenesis, also highlighting the therapeutic potential of inflammatory cytokines-targeted intervention.

## Acknowledgments

We thank all the researchers who provided data on inflammatory factors, hepatic fibrosis, and blood metabolites, as well as the English translators of this paper.

## Author contributions

**Conceptualization:** Liqun Li, Sheng Xie.

**Data curation:** Laian Ge.

**Formal analysis:** Lijian Liu.

**Funding acquisition:** Sheng Xie.

**Investigation:** Jing Yan.

**Methodology:** Liqun Li, Qian Liu, Yifeng Pan.

**Project administration:** Laian Ge.

**Resources:** Qian Liu, Yifeng Pan.

**Software:** Jing Yan.

**Supervision:** Lijian Liu, Sheng Xie.

**Validation:** Liqun Li.

**Visualization:** Jing Yan.

**Writing – original draft:** Liqun Li, Jing Yan, Qian Liu, Laian Ge, Yifeng Pan, Bingjie Han, Chunmei Wang, Xiaomei Tang, Sheng Xie.

**Writing – review & editing:** Lijian Liu, Sheng Xie.

## Supplementary Material



## References

[1] Devarbhavi H Asrani SK Arab JP Nartey YA Pose E Kamath PS. Global burden of liver disease: 2023 update. J Hepatol. 2023;79:516–37. 36990226 10.1016/j.jhep.2023.03.017

[2] Roehlen N Crouchet E Baumert TF. Liver fibrosis: mechanistic concepts and therapeutic perspectives. Cells. 2020;9:875. 32260126 10.3390/cells9040875 PMC7226751

[3] Yokosaki Y Nishimichi N. New therapeutic targets for hepatic fibrosis in the integrin family, α8β1 and α11β1, induced specifically on activated stellate cells. Int J Mol Sci. 2021;22:12794. 34884600 10.3390/ijms222312794 PMC8657911

[4] Berumen J Baglieri J Kisseleva T Mekeel K. Liver fibrosis: pathophysiology and clinical implications. WIREs Mech Dis. 2021;13:e1499. 32713091 10.1002/wsbm.1499 PMC9479486

[5] Man S Deng Y Ma Y. Prevalence of liver steatosis and fibrosis in the general population and various high-risk populations: a nationwide study with 5.7 million adults in China. Gastroenterology. 2023;165:1025–40. 37380136 10.1053/j.gastro.2023.05.053

[6] Abdullahi A Stanojcic M Parousis A Patsouris D Jeschke MG. Modeling acute ER stress in vivo and in vitro. Shock. 2017;47:506–13. 27755507 10.1097/SHK.0000000000000759 PMC5348263

[7] Lee Y-S Funk LH Lee JK Bunge MB. Macrophage depletion and Schwann cell transplantation reduce cyst size after rat contusive spinal cord injury. Neural Regen Res. 2018;13:684–91. 29722321 10.4103/1673-5374.230295 PMC5950679

[8] Zhang Z Wang L Lin Z. Dietary inflammatory index and risk of non-alcoholic fatty liver disease and advanced hepatic fibrosis in US adults. Front Nutr. 2023;10:1102660. 36761224 10.3389/fnut.2023.1102660 PMC9907028

[9] Weiskirchen R. Hepatoprotective and anti-fibrotic agents: it’s time to take the next step. Front Pharmacol. 2015;6:303. 26779021 10.3389/fphar.2015.00303 PMC4703795

[10] Tacke F Weiskirchen R. An update on the recent advances in antifibrotic therapy. Expert Rev Gastroenterol Hepatol. 2018;12:1143–52. 30261763 10.1080/17474124.2018.1530110

[11] Mehal WZ Schuppan D. Antifibrotic therapies in the liver. Semin Liver Dis. 2015;35:184–98. 25974903 10.1055/s-0035-1550055 PMC5743222

[12] Xiang M Liu T Tian C. Kinsenoside attenuates liver fibro-inflammation by suppressing dendritic cells via the PI3K-AKT-FoxO1 pathway. Pharmacol Res. 2022;177:106092. 35066108 10.1016/j.phrs.2022.106092 PMC8776354

[13] Ke M-Y Xu T Fang Y. Liver fibrosis promotes immune escape in hepatocellular carcinoma via GOLM1-mediated PD-L1 upregulation. Cancer Lett. 2021;513:14–25. 33992711 10.1016/j.canlet.2021.05.007

[14] Ma Y Bao Y Wu L. IL-8 exacerbates CCl4-induced liver fibrosis in human IL-8-expressing mice via the PI3K/akt/HIF-1α pathway. Mol Immunol. 2022;152:111–22. 36327908 10.1016/j.molimm.2022.10.011

[15] Zhangdi H-J Su S-B Wang F. Crosstalk network among multiple inflammatory mediators in liver fibrosis. World J Gastroenterol. 2019;25:4835–49. 31543677 10.3748/wjg.v25.i33.4835 PMC6737310

[16] Burgess S Butterworth A Thompson SG. Mendelian randomization analysis with multiple genetic variants using summarized data. Genet Epidemiol. 2013;37:658–65. 24114802 10.1002/gepi.21758 PMC4377079

[17] Emdin CA Khera AV Kathiresan S. Mendelian randomization. JAMA. 2017;318:1925–6. 29164242 10.1001/jama.2017.17219

[18] Davies NM Holmes MV Davey Smith G. Reading mendelian randomisation studies: a guide, glossary, and checklist for clinicians. BMJ. 2018;362:k601. 30002074 10.1136/bmj.k601 PMC6041728

[19] Skrivankova VW Richmond RC Woolf BAR. Strengthening the reporting of observational studies in epidemiology using mendelian randomization: the STROBE-MR statement. JAMA. 2021;326:1614–21. 34698778 10.1001/jama.2021.18236

[20] Kurki MI Karjalainen J Palta P.; FinnGen. FinnGen provides genetic insights from a well-phenotyped isolated population. Nature. 2023;613:508–18. 36653562 10.1038/s41586-022-05473-8 PMC9849126

[21] Zhao JH Stacey D Eriksson N.; Estonian Biobank Research Team. Genetics of circulating inflammatory proteins identifies drivers of immune-mediated disease risk and therapeutic targets. Nat Immunol. 2023;24:1540–51. 37563310 10.1038/s41590-023-01588-w PMC10457199

[22] Chen Y Lu T Pettersson-Kymmer U. Genomic atlas of the plasma metabolome prioritizes metabolites implicated in human diseases. Nat Genet. 2023;55:44–53. 36635386 10.1038/s41588-022-01270-1 PMC7614162

[23] Burgess S Thompson SG; CRP CHD Genetics Collaboration. Avoiding bias from weak instruments in mendelian randomization studies. Int J Epidemiol. 2011;40:755–64. 21414999 10.1093/ije/dyr036

[24] Bowden J Del Greco MF Minelli C. A framework for the investigation of pleiotropy in two-sample summary data mendelian randomization. Stat Med. 2017;36:1783–802. 28114746 10.1002/sim.7221 PMC5434863

[25] Burgess S Thompson SG. Interpreting findings from mendelian randomization using the MR-egger method. Eur J Epidemiol. 2017;32:377–89. 28527048 10.1007/s10654-017-0255-x PMC5506233

[26] Fabiola Del Greco M Minelli C Sheehan NA. Detecting pleiotropy in mendelian randomisation studies with summary data and a continuous outcome. Stat Med. 2015;34:2926–40. 25950993 10.1002/sim.6522

[27] Verbanck M Chen C-Y Neale B Do R. Detection of widespread horizontal pleiotropy in causal relationships inferred from mendelian randomization between complex traits and diseases. Nat Genet. 2018;50:693–8. 29686387 10.1038/s41588-018-0099-7 PMC6083837

[28] Zavala-Solares MR Fonseca-Camarillo G Valdovinos M. Gene expression profiling of inflammatory cytokines in esophageal biopsies of different phenotypes of gastroesophageal reflux disease: a cross-sectional study. BMC Gastroenterol. 2021;21:201. 33941087 10.1186/s12876-021-01707-7 PMC8094498

[29] Caligiuri A Gentilini A Pastore M Gitto S Marra F. Cellular and molecular mechanisms underlying liver fibrosis regression. Cells. 2021;10:2759. 34685739 10.3390/cells10102759 PMC8534788

[30] Martínez-Becerra F Silva D-A Domínguez-Ramírez L. Analysis of the antimicrobial activities of a chemokine-derived peptide (CDAP-4) on pseudomonas aeruginosa. Biochem Biophys Res Commun. 2007;355:352–8. 17307153 10.1016/j.bbrc.2007.01.188

[31] Mendez-Enriquez E García-Zepeda EA. The multiple faces of CCL13 in immunity and inflammation. Inflammopharmacology. 2013;21:397–406. 23846739 10.1007/s10787-013-0177-5

[32] She S Ren L Chen P. Functional roles of chemokine receptor CCR2 and its ligands in liver disease. Front Immunol. 2022;13:812431. 35281057 10.3389/fimmu.2022.812431 PMC8913720

[33] Meng Y Zhao T Zhang Z Zhang D. The role of hepatic microenvironment in hepatic fibrosis development. Ann Med. 2022;54:2830–44. 36399108 10.1080/07853890.2022.2132418 PMC9677987

[34] Mauffray M Domingues O Hentges F Zimmer J Hanau D Michel T. Neurturin influences inflammatory responses and airway remodeling in different mouse asthma models. J Immunol. 2015;194:1423–33. 25595789 10.4049/jimmunol.1402496

[35] Amir M Yu M He P Srinivasan S. Hepatic autonomic nervous system and neurotrophic factors regulate the pathogenesis and progression of non-alcoholic fatty liver disease. Front Med (Lausanne). 2020;7:62. 32175323 10.3389/fmed.2020.00062 PMC7056867

[36] Man J Zhou W Zuo S. TANGO1 interacts with NRTN to promote hepatocellular carcinoma progression by regulating the PI3K/AKT/mTOR signaling pathway. Biochem Pharmacol. 2023;213:115615. 37211171 10.1016/j.bcp.2023.115615

[37] Zhang Z Shang J Yang Q. Exosomes derived from human adipose mesenchymal stem cells ameliorate hepatic fibrosis by inhibiting PI3K/akt/mTOR pathway and remodeling choline metabolism. J Nanobiotechnology. 2023;21:29. 36698192 10.1186/s12951-023-01788-4 PMC9878808

[38] Zhao Y Qu Y Hao C Yao W. PD-1/PD-L1 axis in organ fibrosis. Front Immunol. 2023;14:1145682. 37275876 10.3389/fimmu.2023.1145682 PMC10235450

[39] Miao Y Jiang M Qi L Yang D Xiao W Fang F. BCAP regulates dendritic cell maturation through the dual-regulation of NF-κB and PI3K/AKT signaling during infection. Front Immunol. 2020;11:250. 32133012 10.3389/fimmu.2020.00250 PMC7040100

[40] Tsuchida T Friedman SL. Mechanisms of hepatic stellate cell activation. Nat Rev Gastroenterol Hepatol. 2017;14:397–411. 28487545 10.1038/nrgastro.2017.38

[41] Bai Y-M Liang S Zhou B. Revealing immune infiltrate characteristics and potential immune-related genes in hepatic fibrosis: based on bioinformatics, transcriptomics and q-PCR experiments. Front Immunol. 2023;14:1133543. 37122694 10.3389/fimmu.2023.1133543 PMC10140356

[42] Atorrasagasti C Piccioni F Borowski S. Acceleration of TAA-induced liver fibrosis by stress exposure is associated with upregulation of nerve growth factor and glycopattern deviations. Int J Mol Sci. 2021;22:5055. 34064584 10.3390/ijms22105055 PMC8151393

[43] Wang W-M Xu X-S Miao C-M. Kupffer cell-derived TNF-α triggers the apoptosis of hepatic stellate cells through TNF-R1/caspase 8 due to ER stress. Biomed Res Int. 2020;2020:8035671. 32802876 10.1155/2020/8035671 PMC7421237

[44] Chu X Jin Q Chen H. CCL20 is up-regulated in non-alcoholic fatty liver disease fibrosis and is produced by hepatic stellate cells in response to fatty acid loading. J Transl Med. 2018;16:108. 29690903 10.1186/s12967-018-1490-y PMC5937820

[45] Estep JM Baranova A Hossain N. Expression of cytokine signaling genes in morbidly obese patients with non-alcoholic steatohepatitis and hepatic fibrosis. Obes Surg. 2009;19:617–24. 19280268 10.1007/s11695-009-9814-x

[46] Kar S Paglialunga S Jaycox SH Islam R Paredes AH. Assay validation and clinical performance of chronic inflammatory and chemokine biomarkers of NASH fibrosis. PLoS One. 2019;14:e0217263. 31291245 10.1371/journal.pone.0217263 PMC6619600

[47] Swanson SA Tiemeier H Ikram MA Hernán MA. Nature as a trialist?: Deconstructing the analogy between mendelian randomization and randomized trials. Epidemiology. 2017;28:653–9. 28590373 10.1097/EDE.0000000000000699 PMC5552969

